# Semi-quantitative visual assessment of chest radiography is associated with clinical outcomes in critically ill patients

**DOI:** 10.1186/s12931-019-1201-0

**Published:** 2019-10-12

**Authors:** Stefanie E. Mason, Paul B. Dieffenbach, Joshua A. Englert, Angela A. Rogers, Anthony F. Massaro, Laura E. Fredenburgh, Angelica Higuera, Mayra Pinilla-Vera, Marta Vilas, Raul San Jose Estepar, George R. Washko, Rebecca M. Baron, Samuel Y. Ash

**Affiliations:** 1 Department of Medicine, Division of Pulmonary and Critical Care Medicine, Brigham and Women’s Hospital, 15 Francis Street, Boston, MA 02115 USA; 2 Department of Internal Medicine, Division of Pulmonary, Critical Care and Sleep Medicine, The Ohio State University Wexner Medical Center, 2050 Kenny Road Suite 2200, Columbus, OH 43221 USA; 3 Department of Medicine, Division of Pulmonary, Critical Care Medicine, Stanford University School of Medicine, 300 Pasteur Dr A165, Stanford, CA 94305 USA; 4 Applied Chest Imaging Laboratory, Department of Radiology, Brigham and Women’s Hospital, Harvard Medical School, 1249 Boylston St Room 216, Boston, MA 02215 USA

**Keywords:** Critical illness, Hospital mortality, Intensive care units, Radiography, Severity of illness index

## Abstract

**Background:**

Respiratory pathology is a major driver of mortality in the intensive care unit (ICU), even in the absence of a primary respiratory diagnosis. Prior work has demonstrated that a visual scoring system applied to chest radiographs (CXR) is associated with adverse outcomes in ICU patients with Acute Respiratory Distress Syndrome (ARDS). We hypothesized that a simple, semi-quantitative CXR score would be associated with clinical outcomes for the general ICU population, regardless of underlying diagnosis.

**Methods:**

All individuals enrolled in the Registry of Critical Illness at Brigham and Women’s Hospital between June 2008 and August 2018 who had a CXR within 24 h of admission were included. Each patient’s CXR was assigned an opacification score of 0–4 in each of four quadrants with the total score being the sum of all four quadrants. Multivariable negative binomial, logistic, and Cox regression, adjusted for age, sex, race, immunosuppression, a history of chronic obstructive pulmonary disease, a history of congestive heart failure, and APACHE II scores, were used to assess the total score’s association with ICU length of stay (LOS), duration of mechanical ventilation, in-hospital mortality, 60-day mortality, and overall mortality, respectively.

**Results:**

A total of 560 patients were included. Higher CXR scores were associated with increased mortality; for every one-point increase in score, in-hospital mortality increased 10% (OR 1.10, CI 1.05–1.16, *p* < 0.001) and 60-day mortality increased by 12% (OR 1.12, CI 1.07–1.17, *p* < 0.001). CXR scores were also independently associated with both ICU length of stay (rate ratio 1.06, CI 1.04–1.07, *p* < 0.001) and duration of mechanical ventilation (rate ratio 1.05, CI 1.02–1.07, *p* < 0.001).

**Conclusions:**

Higher values on a simple visual score of a patient’s CXR on admission to the medical ICU are associated with increased in-hospital mortality, 60-day mortality, overall mortality, length of ICU stay, and duration of mechanical ventilation.

## Background

Over one quarter of annual hospital stays in the United States involve an interaction with the intensive care unit (ICU), representing 22% of total hospital costs and 4.1% of national health expenditures [1]. Accurately and rapidly assessing the severity of illness in this population facilitates optimal resource allocation, provision of care, and appropriate counseling of patients and their families.

Currently, these patients are risk stratified by critical illness scoring systems such as the Acute Physiology and Chronic Health Evaluation (APACHE) or the Sequential Organ Failure Assessment (SOFA) [2–6]. Newer data mining and machine learning techniques have been applied for risk stratification [7–9], but both the traditional and the new models require substantial data inputs and a minimum of 6 h of elapsed time. As a result, they are predominately used in research settings and are applied in clinical practice in fewer than 15% of ICU admissions [4].

Respiratory pathology is a major driver of mortality in the ICU, even in the absence of a primary respiratory diagnosis. For example, the proportion of patients who require mechanical ventilation during their ICU stay is 3-fold higher than the proportion of patients admitted for a respiratory condition [10, 11]. Because of this, the majority of patients admitted to the ICU undergo some form of chest imaging, typically a chest radiograph (CXR), early in their hospital course. Prior work has demonstrated that a different semi-quantitative scoring system applied to the CXR is associated with adverse outcomes in ICU patients with Acute Respiratory Distress Syndrome (ARDS) [12].

We hypothesized that a simple, semi-quantitative CXR score reflecting the density and extent of parenchymal opacification would be associated with clinical outcomes for the general ICU population, regardless of underlying diagnosis. We additionally hypothesized that this score would be correlated with plasma biomarkers associated with mortality in critically-ill populations, as well as with measurements of lung weight at autopsy.

## Materials and methods

### Patient population and data acquisition

The Research Registry and Human Sample Repository for the Study of Biology of Critical Illness, abbreviated as the Registry of Critical Illness (RoCI), has been previously described and collects demographic and clinical information as well as blood samples from patients with critical illness at the Brigham and Women’s Hospital (BWH). Patients in the RoCI represent a prospective convenience sample of patients admitted to the ICU. All patients, or their surrogates, provide written informed consent and the study is approved by the Partner’s Institutional Review Board [13, 14].

**Fig. 1:**
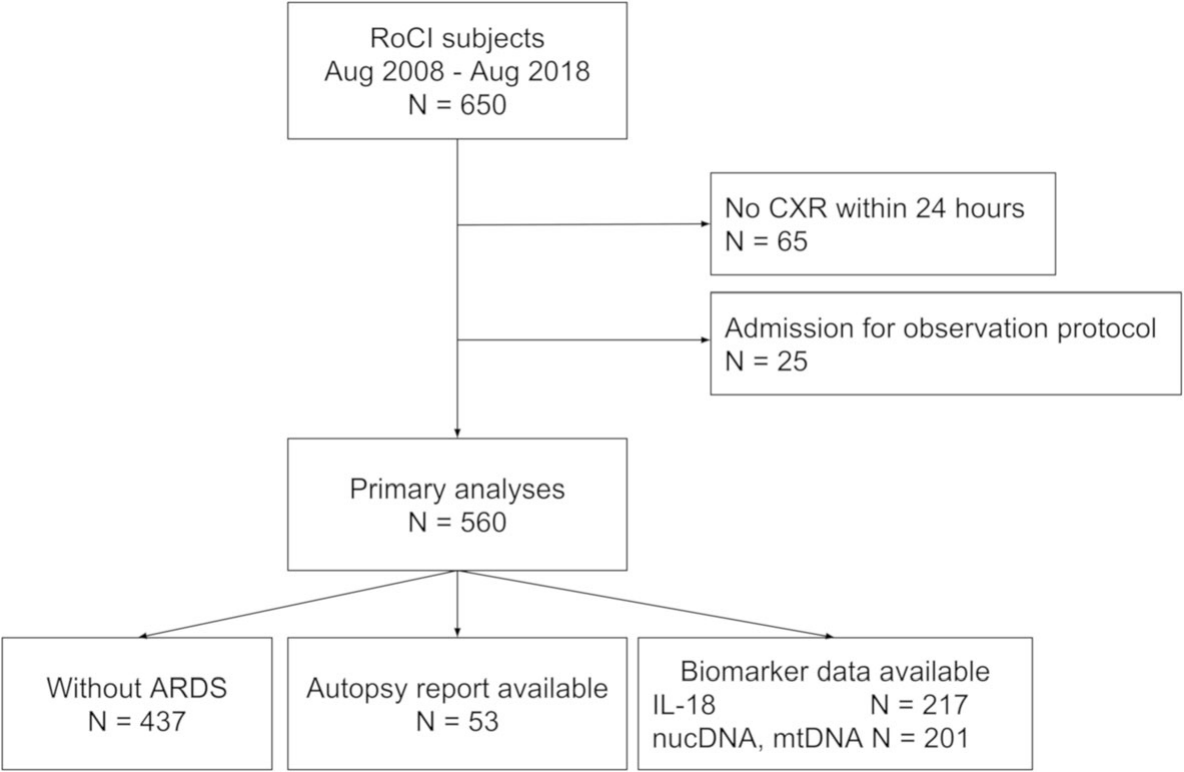
Consort diagram of subjects included in the primary and predefined subgroup analyses. RoCI, Registry of Critical Ilness; CXR, chest radiograph; ARDS, acute respiratory distress syndrome; IL-18, interleukin-18; nucDNA, nuclear DNA; mtDNA, mitochondrial DNA

For this study, we included the subset of patients enrolled between August 2008 and August 2018 (*n* = 650) who had a CXR within 24 h of admission to a medical ICU (*n* = 585). Patients were excluded if their admission was for an observation protocol, such as desensitization to an antibiotic (*n* = 25). Secondary analysis was performed on predefined subsets including those without ARDS (*n* = 437) and those with autopsy information (*n* = 53) (Fig. 1).

Mortality data, including date of death, were obtained from the clinical record and the social security death index. Clinical data was obtained from the electronic medical record. Sepsis was determined using the sepsis-3 criteria and ARDS by the Berlin definition [15, 16]. Immunosuppression was considered untreated hematologic malignancy, active chemotherapy or other chronic immunosuppressive medication, including prednisone at doses of 20 mg per day or more. The presence of congestive heart failure (CHF) or chronic obstructive pulmonary disease (COPD) was determined by review of medical history and problem lists. All diagnoses were determined by consensus of a group of two or more pulmonary and critical care physicians.

### Radiograph scoring

**Fig. 2:**
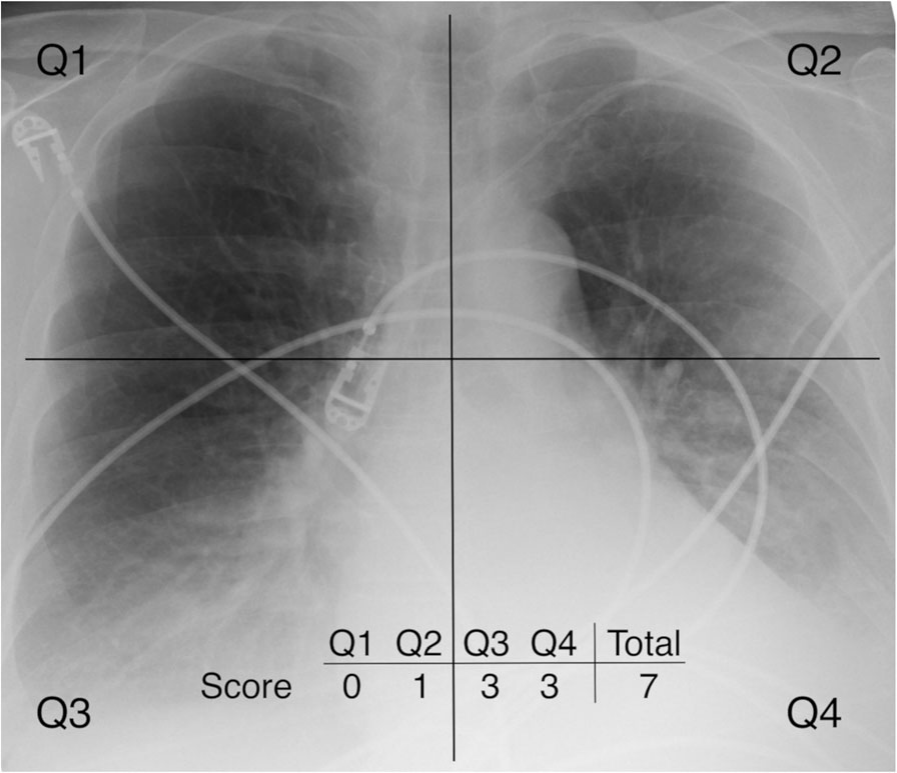
An example of a scored chest radiograph. Each image is divided into four quadrants (Q) with each quadrant assigned a score from 0 to 4. The total score, representing the sum of the four quadrants, was used as a predictor in our clinical outcomes models

Since the inception of the RoCI, the chest radiographs of all the participants have been evaluated and scored using an independently developed scoring system. To calculate a score for each radiograph, the patient’s first portable CXR upon acceptance to the ICU was divided into four quadrants; two in each of the right and left lungs. The upper and lower quadrants for each lung were determined by the level of the carina. Each quadrant was assigned a score from 0 to 4 with 0 = normal, 1 = hazy opacification of less than half the quadrant, 2 = dense opacification of less than half the quadrant, 3 = hazy opacification of more than half the quadrant, and 4 = dense opacification of more than half the quadrant. Opacification was considered dense if the rib margins were obscured by the infiltrate. The total score, ranging from 0 to 16, was the sum of the four quadrants (Fig. 2). If the subject initially presented to another facility and the only image available for the first 24 h was obtained there, then the image from the facility of presentation was used (*n* = 6). No radiographs were excluded for positioning or exposure reasons.

Each radiograph was scored based on the consensus of 2–4 pulmonary and critical care physicians. For the purpose of inter-rater reliability, 40% (*n* = 222) of the radiographs, chosen randomly, were scored by a consensus of two different pulmonary and critical care physicians who were blinded to the original score. In addition, 18% (*n* = 98) of the radiographs, chosen randomly, were scored by a radiologist who was blinded to the original scores.

For the patients who underwent an autopsy (*n* = 53), a CXR within 24 h of death was scored by a pulmonary and critical care physician who was blinded to both the lung weights recorded in the autopsy report and the admission CXR score.

### Statistical analysis

Summary statistics are reported using medians and interquartile ranges (IQR) or frequencies and percentages as appropriate. The reproducibility of the CXR score between independent reviewers was assessed using a two-way mixed consistency, single-measures intraclass correlation and Bland-Altman plots were used to visualize the agreement.

Associations between the CXR score (measured continuously) and in-hospital mortality, 60-day mortality, length of stay, duration of mechanical ventilation, and overall survival were evaluated using multivariable logistic, negative binomial, and Cox regressions respectively. These analyses were adjusted for APACHE II score, age, race, sex, a history of COPD, a history of CHF, and immunosuppression status. All of the covariates were assessed using the Schoenfeld residuals method and none were found to violate the proportional hazards assumption [17].

In order to evaluate for a threshold effect, the regression analyses were repeated using categorical CXR score quartiles with Bonferroni correction for multiple comparisons. Kaplan-Meier curves and the log rank test were used to visualize and assess for differences in overall survival by quartile and trends in ICU length of stay and duration of mechanical ventilation by quartile were visualized using boxplots and assessed using the Jonckheere-Terpstra trend test.

Univariable and bivariable receiver operating curves (ROC) were generated for total CXR score, APACHE II score, and SOFA score as predictors of in-hospital mortality. The area under the ROC curve (AUC) was compared across univariable ROC curves using the Delong method and between nested ROC curves using the Heller method [18, 19].

The association between CXR score and individual lung weights at autopsy was evaluated with multivariable linear regression adjusted for height and sex. Spearman correlation was used to assess associations between the continuous CXR score and serum biomarkers previously measured and found to be associated with sepsis in the RoCI and other cohorts (interleukin-18 (IL-18), nuclear DNA (nucDNA), and mitochondrial DNA (mtDNA)) [13, 14].

All statistical tests were two-sided. A *p*-value of < 0.05 was considered to indicate statistical significance. All of the analyses were performed using R version 3.5.1.

## Results

### Clinical outcomes

The characteristics of the 560 patients included in the analysis are given in Table 1. In general, patients were predominately Caucasian (77.9%, *n* = 300) and admitted for sepsis (69.5%, *n* = 389). There were 286 intubated patients with a median duration of mechanical ventilation of 5 days (IQR 3–10). The median length of ICU stay was 5 days (IQR 3–8). Patient mortality data was available for a median post-discharge period of 12.1 months (IQR 0.7–59.6 months). Overall mortality in the cohort was 66% (*n* = 369) inclusive of the 28% (*n* = 159) mortality during index hospitalization.

**Table 1:** Characteristics of the study population. Data are median and interquartile range (IQR) or number and percent as appropriate. APACHE, acute physiology and chronic health evaluation; ARDS, acute respiratory distress syndrome; CHF, congestive heart failure; COPD, chronic obstructive pulmonary disease

Characteristic	Population (*n* = 560)
Age (years)	60 (47, 70)
Male	300 (53.6%)
Caucasian	436 (77.9%)
Immunosuppressed	205 (36.6%)
APACHE II	25 (20, 31)
Intubated	286 (51.1%)
ARDS	123 (22.0%)
Sepsis	389 (69.5%)
History of CHF	47 (8.4%)
History of COPD	90 (16.1%)
In-hospital mortality	159 (28.4%)
60-day mortality	185 (33.0%)

The median CXR score was 7 (IQR 3–11) resulting in categorical quartiles of 0–2, 3–6, 7–11, and 12–16. Score determination took an average of 60–90 s once the radiograph was accessed. Of the 222 CXR scored by both critical care reviewer groups, only 23 scores differed by more than two points. The intraclass correlation (ICC) between the critical care reviewers was 0.93 (CI 0.91–0.95), indicating substantial agreement (Additional file 1: Figure S1). The ICC including the radiology reader was 0.85 (0.77–0.90). The results of the regressions for in-hospital and 60-day mortality did not differ within the reported significant digits regardless of which reader’s scores were used. Similarly, the results of the regression analyses did not change if the outside hospital CXR (*n* = 6) were excluded.

**Fig. 3:**
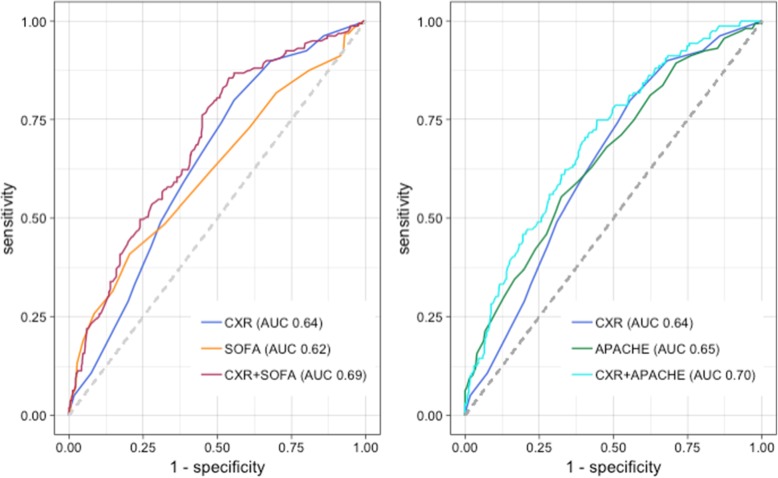
Receiver operating curves (ROC) for univariable and bivariable predictors of in-hospital mortality. AUC area under the curve; CXR chest radiograph; SOFA sequential organ failure assessment; APACHE acute physiology and chronic health evaluation

The total CXR score had similar discriminative ability for in-hospital mortality in this cohort as the APACHE II and SOFA scores as measured by the area under the ROC (AUC) (Fig. 3). The AUC for the total CXR score was 0.64, which was not significantly different from the SOFA (0.62, *p* = 0.574) or APACHE (0.65, *p* = 0.711). Adding the CXR score to either the APACHE II or SOFA predictor increased the AUC to 0.70 (*p* < 0.001) and 0.69 (*p* < 0.001) respectively, which represented an improvement compared to either illness score alone.

**Fig. 4:**
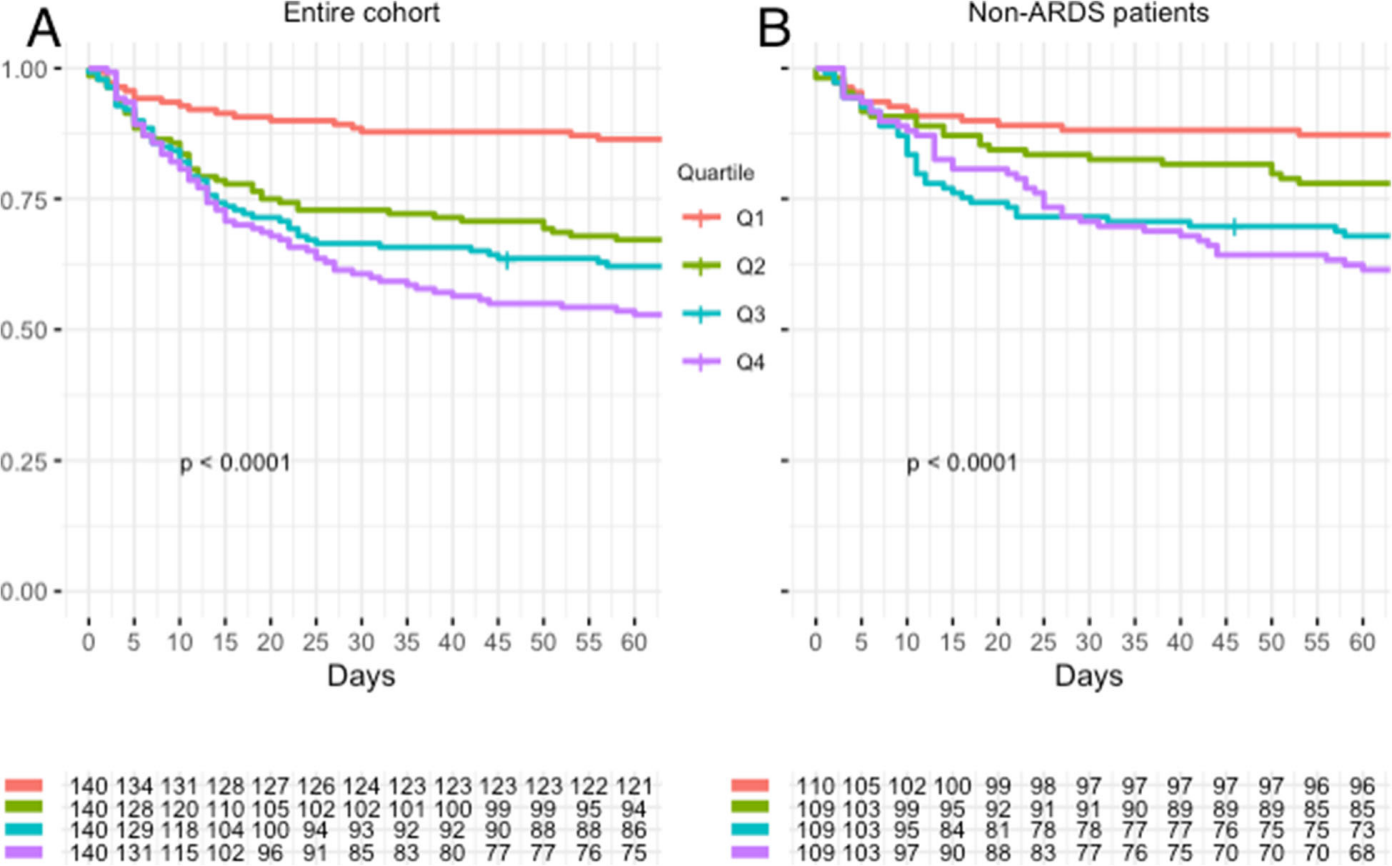
60-day survival stratified by chest radiograph score quartile for the entire cohort (panel **a**) and excluding subjects with Acute Respiratory Distress Syndrome (ARDS) (panel **b**)

Higher CXR scores were independently associated with increased mortality; for every one-point increase in score, in-hospital mortality increased 10% (OR 1.10, CI 1.05–1.16, *p* < 0.001) and 60-day mortality increased by 12% (OR 1.12, CI 1.07–1.17, *p* < 0.001). When stratified by quartile, individuals in quartiles 2, 3, and 4 all had higher mortality than those in quartile 1, but the differences between quartiles 2, 3, and 4 were not significant for either in-hospital or 60-day mortality (Fig. 4, Additional file 1: Tables S1 & S2).

Similar findings were present in patients without ARDS. In that subgroup, a one-point increase in CXR score was associated with 8% increase in in-hospital mortality (OR 1.08, CI 1.02–1.15, *p* = 0.009), and a 12% increase in 60-day mortality (OR 1.12, CI 1.06–1.19, *p* < 0.001). As in the entire cohort, in the subgroup without ARDS, those individuals in quartiles 2, 3 and 4 had higher overall mortality than those in quartile 1, but the differences between quartiles 2, 3, and 4 were not significant (Fig. 4, Additional file 1: Tables S1 & S2).

Overall mortality demonstrated a similar trend; with every one-point increase in CXR score there was a 6% increase in mortality (HR 1.06, CI 1.03–1.08, *p* < 0.001) for the entire cohort and a 7% higher overall mortality (HR 1.07, CI 1.04–1.10, *p* < 0.001) in the subgroup without ARDS (Additional file 1: Figure S2 and Additional file 1: Table S3). None of the mortality measures were significant in the subgroup of patients with ARDS (Additional file 1: Table S4).

**Fig. 5:**
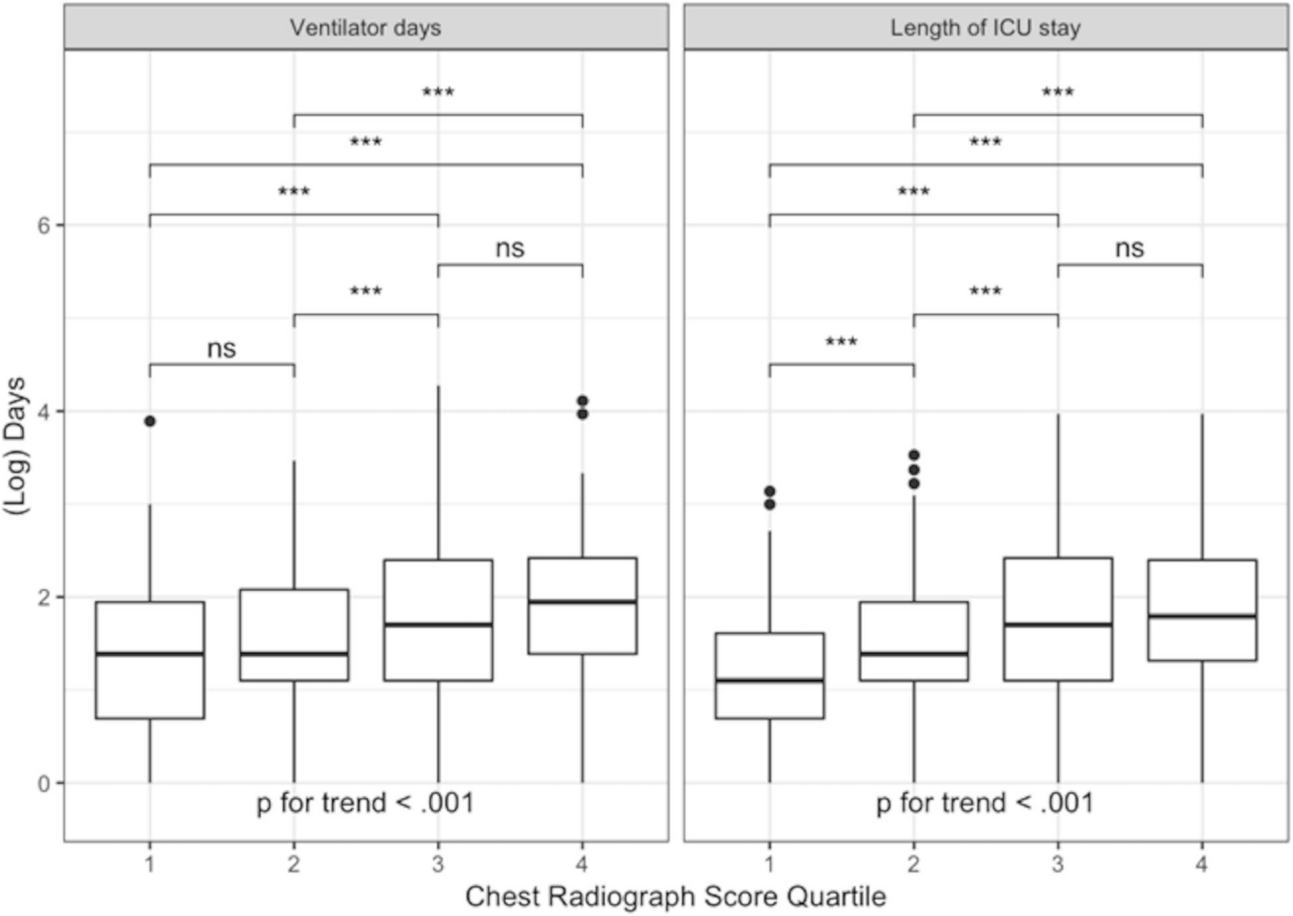
Duration of mechanical ventilation and length of intensive care unit admission stratified by chest radiograph score quartile. Presence of trend assessed using the Jonkheere Terpstra test and pairwise comparisons evaluated using negative binomial regression with Bonferroni correction for multiple comparisons. (***) indicates significance by adjusted *p*-value, (ns) represents adjusted *p*-values that were not significant

CXR scores were independently associated with both length of ICU stay and duration of mechanical ventilation (Fig. 5). For every one-point increase in CXR score, the length of ICU stay increased by 0.40 days (rate ratio 1.06, CI 1.04–1.07, *p* < 0.001) and the duration of mechanical ventilation increased 0.37 days (rate ratio 1.05, CI 1.02–1.07, *p* < 0.001). When stratified by quartile, those individuals in the higher CXR score quartiles had longer ICU stays and durations of mechanical ventilation than those in lower CXR score quartiles (*p* < 0.001 for trend for both), but there was not a significant difference between quartiles 3 and 4 with regard to length of ICU stay, nor a significant difference between quartiles 1 and 2 or between quartiles 3 and 4 for the duration of mechanical ventilation (Additional file 1: Tables S5 & S6).

### Lung weights

A total of 53 patients underwent autopsy resulting in 105 individually measured lung weights (1 subject had undergone pneumonectomy). The median weight was 910 g (IQR 649–1090 g). The CXR score was correlated with lung weight such that for every one-point increase in CXR score, the lung weight increased by 49 g (β = 49.1, CI 18.8–79.5, *p* = 0.002) (Additional file 1: Figure S3).

### Critical illness biomarkers

Plasma IL-18 levels were available for 217 patients; nucDNA and mtDNA data were available for 201 (Fig. 1). As shown in Additional file 1: Figure S3, circulating mtDNA and nucDNA were significantly associated with CXR score (*r* = 0.23, *p* = 0.002 and *r* = 0.20, *p* = 0.008 respectively). IL-18 was not significantly associated with CXR score (*r* = 0.09, *p* = 0.219).

## Discussion

In this study we found that a simple, semi-quantitative visual CXR score on admission to the ICU predicts clinical outcomes in a general medical ICU population. In addition, it appears that there may be a threshold effect such that mild abnormalities on admission CXR are associated with significantly worse clinical outcomes. We further found that the same scoring system is associated with plasma-based biomarkers of critical illness and, when applied to a CXR within 24 h of death, is associated with lung weight at autopsy.

CXR scoring systems have been explored as predictors of outcome in a variety of respiratory pathologies, however these studies generally included only patients with specific respiratory diagnoses and many were designed for outpatient longitudinal care, limiting their generalizability [12, 20–23] . This study extends the use of CXR scoring systems to a broader ICU population and supports the association of worse clinical outcomes, such as increased in-hospital and 60-day mortality, with higher CXR scores, regardless of the underlying diagnosis.

The CXR score used in this study was able to discriminate in-hospital mortality as well as the APACHE II score and better than the SOFA score, as measured by the area under the ROC. Further, the addition of the CXR score to either the SOFA or APACHE II score improved the discrimination, suggesting the CXR score provides novel information not fully captured by these existing scoring systems. Compared to the time and volume of data required to calculate an APACHE or SOFA score, the CXR score represents an efficient potential alternative screening tool for identifying high-risk ICU patients, though further study is required before it could be introduced clinically.

Both our study, and previous work, found a potential threshold effect with regard to mortality. In our study, given the stepwise increase in odds of death by quartile, it is possible that with a larger sample size this threshold effect would be eliminated. Notably, the location of the potential threshold differed between the two studies; with ours suggesting an inflection after quartile one and theirs at the median [12].. This likely reflects the differences in the scoring systems as well as in the populations to which they were applied. The prior study was limited to ARDS patients enrolled in the Fluid and Catheter Treatment Trial, a randomized, controlled trial that excluded patients with comorbid conditions limiting life-expectancy, which significantly reduced heterogeneity [24]. That we were unable to replicate their findings specifically in the subgroup with ARDS is likely due to our limited sample size of this subgroup.

Our study has several strengths. These include the cohort size and heterogeneity as well as the prolonged duration of over which mortality could be assessed. The scoring system used is uncomplicated, noninvasive, reproducible, and rapidly calculated from routinely obtained clinical testing and thus could be easily employed at the bedside in the ICU. Our use of clinically-acquired images assessed by ICU personnel, including images from outside facilities or images that may have been compromised by patient rotation or suboptimal exposure, demonstrates the score’s discriminative ability under practical circumstances.

In addition, the correlations we found with both plasma-based biomarkers associated with critical illness and with lung weights at autopsy suggest the existence of biologic underpinnings to our findings. Elevated IL-18 levels have been demonstrated in patients with sepsis and ARDS compared to ICU controls [14, 25, 26]. Similarly, damage associated molecular patterns, such as mtDNA and nucDNA have been associated with mortality in critically ill patients [27, 28]. While the strength of the correlation between biomarkers and the CXR score is moderate, this is likely because the correlation between biomarkers and critical illness itself has been variable [29]. Further, because our cohort was comprised predominately of sepsis, we chose biomarkers studied in septic critical illness; however, the correlation for a given biomarker is likely to be limited given the heterogeneity of diagnoses admitted to the ICU.

Our study also has several limitations. For example, subgroups in our cohort were not large enough to explore the relationship between the variety of admission diagnoses and mortality with granularity. Our non-ARDS subgroup was comprised predominately of septic patients and thus likely underrepresents diagnoses such as cardiogenic shock or COPD exacerbation, which may limit generalizability. Additionally, the population in our study is derived from a single, tertiary care institution with a high proportion of immunosuppressed patients. While immunosuppression was controlled for in the regression models, our survival outcomes may not be typical of a community ICU setting. Indeed, our median CXR score was comparable to, and our 60-day mortality was higher than, that of a prior study composed entirely of ARDS patients. It will be important to validate our findings in other general ICU populations that include a range of illness severity and comorbid conditions.

## Conclusions

Even small increases in a simple, semi-quantitative visual score of the opacification of a patient’s chest radiograph on admission to the medical ICU are associated with increased mortality, length of ICU stay, and duration of mechanical ventilation. Although replication and further work are needed, these findings suggest that the presence of mild abnormalities on ICU admission CXR could be used as a screening tool to identify patients at the highest risk for adverse outcomes.

## Supplementary information

**Additional file 1: Figure S1.** Scatterplot (panel A) and Bland Altman plot (panel B) demonstrating good agreement between reviewers (ICC 0.93, CI 0.91–0.95). **Figure S2.** Kaplan Meier curves depicting overall mortality stratified by chest radiograph quartile for the entire cohort (panel A) and excluding ARDS patients (panel B). **Figure S3.** Scatterplots depicting the correlation between serum biomarkers of critical illness and total chest radiograph scores. **Table S1.** The odds ratios for 60-day mortality by chest radiograph score quartile. **Table S2.** The odds ratios for in-hospital mortality by chest radiograph score quartile. **Table S3.** The odds ratios for overall mortality by chest radiograph score quartile. **Table S4.** In-hospital, 60-day, and overall mortality for patients with Acute Respiratory Distress Syndrome. **Table S5.** Odds ratios for duration of stay in the intensive care unit by chest radiograph score quartile. **Table S6.** Odds ratios for duration of mechanical ventilation for intubated patients (*n* = 286) by chest radiograph score quartile.

## Data Availability

The datasets analysed during the current study are not publicly available as they contain information that could compromise patient privacy, but are available from the corresponding author on reasonable request.

## Abbreviations

APACHE: Acute physiology and chronic health evaluation
ARDS: Acute respiratory distress syndrome
AUC: Area under the curve
CI: Confidence interval
CXR: Chest radiograph
HR: Hazard ratio
ICU: Intensive care unit
IL-18: Intereukin-18
IQR: Interquartile range
mtDNA: Mitochondrial DNA
nucDNA: Nuclear DNA
OR: Odds ratio
ROC: Receiver operating curve
RoCI: Registry of critical illness
