# Supplementary material for: Semi-quantitative visual assessment of chest radiography is associated with clinical outcomes in critically ill patients
Source: Respir Res. 2019 Oct 12;20:218. doi: 10.1186/s12931-019-1201-0 (PMC6790038; doi:10.1186/s12931-019-1201-0)
Supplement: Supplementary file 1 — Additional file 1: Figure S1. Scatterplot (panel A) and Bland Altman plot (panel B) demonstrating good agreement between reviewers (ICC 0.93, CI 0.91–0.95). Figure S2. Kaplan Meier curves depicting overall mortality stratified by chest radiograph quartile for the entire cohort (panel A) and excluding ARDS patients (panel B). Figure S3. Scatterplots depicting the correlation between serum biomarkers of critical illness and total chest radiograph scores. Table S1. The odds ratios for 60-day mortality by chest radiograph score quartile. Table S2. The odds ratios for in-hospital mortality by chest radiograph score quartile. Table S3. The odds ratios for overall mortality by chest radiograph score quartile. Table S4. In-hospital, 60-day, and overall mortality for patients with Acute Respiratory Distress Syndrome. Table S5. Odds ratios for duration of stay in the intensive care unit by chest radiograph score quartile. Table S6. Odds ratios for duration of mechanical ventilation for intubated patients (n = 286) by chest radiograph score quartile. [file 12931_2019_1201_MOESM1_ESM.docx]

**Figure S1**. Scatterplot (panel A) and Bland Altman plot (panel B) demonstrating good agreement between pulmonary and critical care reviewers (ICC 0.93, CI 0.91-0.95).


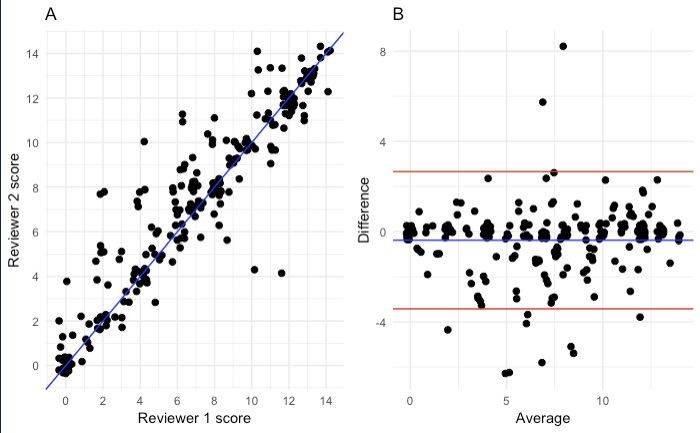


**Figure S2.** Overall mortality stratified by chest radiograph score quartile for the entire cohort (panel A) and excluding subjects with Acute Respiratory Distress Syndrome (ARDS) (panel B).

A

B


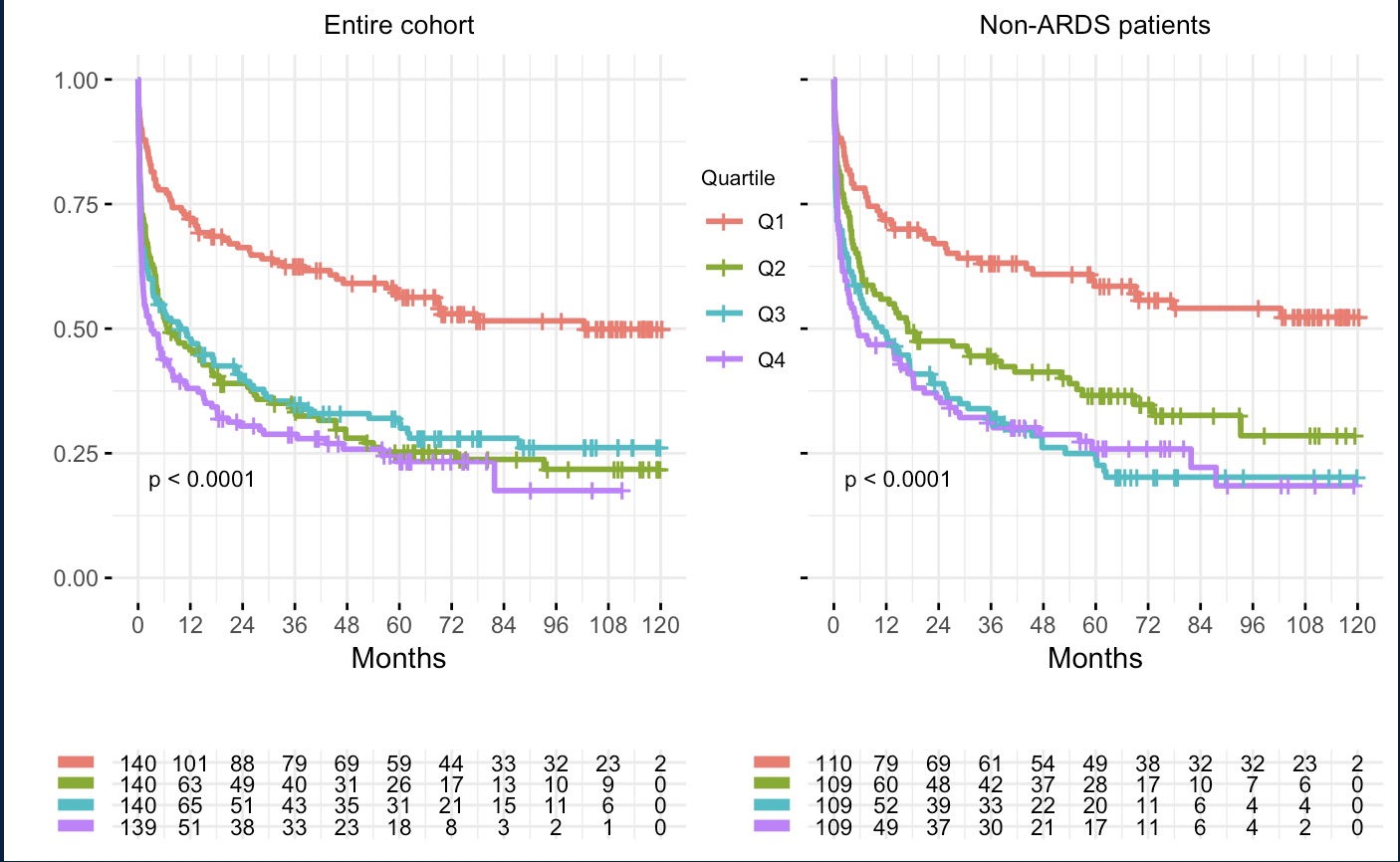


**Figure S3.** Association between chest radiograph total score and biomarkers of critical illness and lung weights on autopsy.


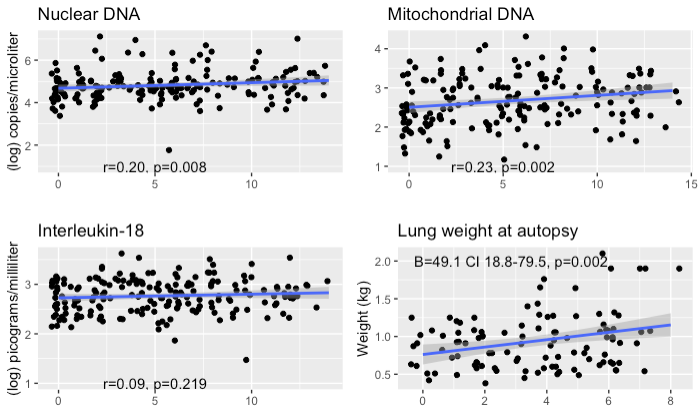


**Table S1.** 60-day mortality by chest radiograph score quartile. P-values have been adjusted with the

Bonferroni correction for multiple testing.

| Entire cohort (n = 560) | | | | | | | | |
| --- | --- | --- | --- | --- | --- | --- | --- | --- |
| Quartile | Odds Ratio  (Confidence Interval) | Adjusted  p-value | Odds Ratio  (Confidence Interval) | Adjusted  p-value | Odds Ratio  (Confidence Interval) | Adjusted  p-value | Odds Ratio  (Confidence Interval) | Adjusted  p-value |
| 1 | reference |  | 0.36 (0.19-0.68) | 0.011 | 0.32 (0.16-0.59) | 0.003 | 0.22 (0.11-0.41) | <0.001 |
| 2 | 2.78 (1.48-5.39) | 0.011 | reference |  | 0.88 (0.51-1.51) | >0.999 | 0.61 (0.36-1.03) | 0.407 |
| 3 | 3.16 (1.69-6.10) | 0.003 | 1.14 (0.66-1.95) | >0.999 | reference |  | 0.70 (0.41-1.17) | >0.999 |
| 4 | 4.55 (2.45-8.72) | <0.001 | 1.63 (0.97-2.78) | 0.407 | 1.44 (0.86-2.43) | >0.999 | reference |  |
| Excluding Acute Respiratory Distress Syndrome patients (n = 437) | | | | | | | | |
| 1 | reference |  | 0.57 (0.26-1.22) | 0.940 | 0.35 (0.17-0.71) | 0.028 | 0.25 (0.12-0.50) | 0.001 |
| 2 | 1.74 (0.82-3.82) | 0.940 | reference |  | 0.61 (0.32-1.16) | 0.824 | 0.43 (0.22-0.81) | 0.063 |
| 3 | 2.84 (1.40-6.00) | 0.028 | 1.63 (0.86-3.14) | 0.824 | reference |  | 0.70 (0.38-1.27) | >0.999 |
| 4 | 4.07 (2.00-8.66) | 0.001 | 2.34 (1.23-4.53) | 0.063 | 1.43 (0.79-2.63) | >0.999 | reference |  |

**Table S2**. In-hospital mortality by chest radiograph score quartile. P-values have been adjusted with the

Bonferroni correction for multiple testing.

| Entire cohort (n = 560) | | | | | | | | |
| --- | --- | --- | --- | --- | --- | --- | --- | --- |
| Quartile | Odds Ratio  (Confidence Interval) | Adjusted  p-value | Odds Ratio  (Confidence Interval) | Adjusted  p-value | Odds Ratio  (Confidence Interval) | Adjusted  p-value | Odds Ratio  (Confidence Interval) | Adjusted  p-value |
| 1 | reference |  | 0.38 (0.19-0.74) | 0.032 | 0.30 (0.14-0.54) | 0.001 | 0.27 (0.13-0.52) | 0.001 |
| 2 | 2.66 (1.36-5.40) | 0.032 | reference |  | 0.74 (0.43-1.30) | >0.999 | 0.71 (0.41-1.23) | >0.999 |
| 3 | 3.57 (1.85-7.19) | 0.001 | 1.34 (0.77-2.35) | >0.999 | reference |  | 0.96 (0.56-1.64) | >0.999 |
| 4 | 3.72 (1.93-7.45) | 0.001 | 1.40 (0.81-2.43) | >0.999 | 1.04 (0.61-1.77) | >0.999 | reference |  |
| Excluding Acute Respiratory Distress Syndrome patients (n = 437) | | | | | | | | |
| 1 | reference |  | 0.66 (0.29-1.47) | >0.999 | 0.35 (0.16-0.73) | 0.039 | 0.42 (0.19-0.90) | 0.168 |
| 2 | 1.53 (0.68-3.50) | >0.999 | reference |  | 0.54 (0.27-1.06) | 0.458 | 0.64 (0.32-1.29) | >0.999 |
| 3 | 2.83 (1.36-6.16) | 0.039 | 1.86 (0.94-3.73) | 0.458 | reference |  | 1.19 (0.63-2.26) | >0.999 |
| 4 | 2.37 (1.11-5.25) | 0.168 | 1.56 (0.77-3.17) | >0.999 | 0.84 (0.44-1.58) | >0.999 | reference |  |

**Table S3.** Overall mortality by chest radiograph score quartile. P-values have been adjusted with the

Bonferroni correction for multiple testing.

| Entire cohort (n = 560) | | | | | | | | |
| --- | --- | --- | --- | --- | --- | --- | --- | --- |
| Quartile | Hazard Ratio  (Confidence Interval) | Adjusted  p-value | Hazard Ratio  (Confidence Interval) | Adjusted  p-value | Hazard Ratio  (Confidence Interval) | Adjusted  p-value | Hazard Ratio  (Confidence Interval) | Adjusted  p-value |
| 1 | reference |  | 0.49 (0.35-0.67) | <0.001 | 0.51 (0.37-0.70) | <0.001 | 0.46 (0.33-0.63) | <0.001 |
| 2 | 2.06 (1.49-2.84) | <0.001 | reference |  | 1.05 (0.79-1.40) | >0.999 | 0.94 (0.71-1.25) | >0.999 |
| 3 | 1.97 (1.42-2.72) | <0.001 | 0.95 (0.72-1.27) | >0.999 | reference |  | 0.90 (0.68-1.19) | >0.999 |
| 4 | 2.19 (1.59-3.01) | <0.001 | 1.06 (0.80-1.40) | >0.999 | 1.11 (0.84-1.47) | >0.999 | reference |  |
| Excluding Acute Respiratory Distress Syndrome patients (n = 437) | | | | | | | | |
| 1 | reference |  | 0.56 (0.38-0.81) | 0.015 | 0.43 (0.30-0.62) | <0.001 | 0.40 (0.27-0.58) | <0.001 |
| 2 | 1.80 (1.23-2.64) | 0.015 | reference |  | 0.79 (0.56-1.08) | 0.800 | 0.72 (0.51-1.00) | 0.315 |
| 3 | 2.31 (1.60-3.33) | <0.001 | 1.28 (0.93-1.78) | 0.800 | reference |  | 0.92 (0.67-1.27) | >0.999 |
| 4 | 2.51 (1.72-3.65) | <0.001 | 1.39 (1.00-1.94) | 0.315 | 1.08 (0.79-1.49) | >0.999 | reference |  |

**Table S4**. In-hospital, 60-day, and overall

mortality for patients with Acute Respiratory Distress Syndrome.

|  | | Odds Ratio (Confidence Interval) | p-value |
| --- | --- | --- | --- |
| In-hospital mortality | | 1.08 (0.95-1.23) | 0.227 |
| 60-day mortality | | 1.07 (0.94-1.22) | 0.308 |
|  | | Hazard Ratio (Confidence Interval) | p-value |
| Overall mortality | | 0.99 (0.93-1.06) | 0.840 |
|  | Quartile 1 | reference |  |
|  | Quartile 2 | 0.90 (0.48-1.71) | 0.750 |
|  | Quartile 3 | 0.93 (0.49-1.75) | 0.814 |
|  | Quartile 4 | 0.89 (0.47-1.67) | 0.710 |

**Table S5**. Duration of stay in the intensive care unit by chest radiograph score quartile. P-values have been adjusted with the Bonferroni correction for multiple testing.

| Quartile | Rate Ratio  (Confidence Interval) | Adjusted  p-value | Rate Ratio  (Confidence Interval) | Adjusted  p-value | Rate Ratio  (Confidence Interval) | Adjusted  p-value | Rate Ratio  (Confidence Interval) | Adjusted  p-value |
| --- | --- | --- | --- | --- | --- | --- | --- | --- |
| 1 | reference |  | 0.72 (0.60-0.85) | 0.001 | 0.56 (0.47-0.66) | <0.001 | 0.52 (0.44-0.62) | <0.001 |
| 2 | 1.40 (1.17-1.66) | 0.001 | reference |  | 0.78 (0.66-0.92) | 0.019 | 0.73 (0.62-0.86) | 0.001 |
| 3 | 1.79 (1.50-2.13) | <0.001 | 1.28 (1.09-1.51) | 0.019 | reference |  | 0.93 (0.79-1.09) | >0.999 |
| 4 | 1.92 (1.62-2.29) | <0.001 | 1.38 (1.17-1.62) | 0.001 | 1.08 (0.92-1.26) | >0.999 | reference |  |

**Table S6.** Duration of mechanical ventilation for intubated patients (n = 286) by chest radiograph score quartile. P-values have been adjusted with the Bonferroni correction for multiple testing.

| Quartile | Rate Ratio  (Confidence Interval) | Adjusted  p-value | Rate Ratio  (Confidence Interval) | Adjusted  p-value | Rate Ratio  (Confidence Interval) | Adjusted  p-value | Rate Ratio  (Confidence Interval) | Adjusted  p-value |
| --- | --- | --- | --- | --- | --- | --- | --- | --- |
| 1 | reference |  | 0.98 (0.70-1.38) | >0.999 | 0.65 (0.47-0.90) | 0.043 | 0.64 (0.47-0.88) | 0.025 |
| 2 | 1.02 (0.73-1.43) | >0.999 | reference |  | 0.66 (0.50-0.87) | 0.016 | 0.65 (0.50-0.85) | 0.008 |
| 3 | 1.54 (1.11-2.14) | 0.043 | 1.51 (1.15-1.99) | 0.016 | reference |  | 0.98 (0.77-1.26) | >0.999 |
| 4 | 1.57 (1.14-2.14) | 0.025 | 1.54 (1.18-2.00) | 0.008 | 1.02 (0.79-1.30) | >0.999 | reference |  |
